# Suicidal Risk and Associated Factors Among Elderly Psychiatric Patients

**DOI:** 10.1192/j.eurpsy.2026.11591

**Published:** 2026-07-31

**Authors:** N. Smaoui, M. Barkallah, S. Omri, M. Maalej, R. Feki, I. Gassara, N. Charfi, L. Zouari

**Affiliations:** Psychiatry Department “C”, Hedi Chaker Hospital, Sfax, Tunisia

## Abstract

**Introduction:**

Suicide in older adults is a growing public health concern, especially among those receiving psychiatric care. A comprehensive understanding of the clinical, psychosocial, and personality-related determinants of suicidal risk in this population is crucial for improving early detection and targeted prevention strategies.

**Objectives:**

This study aimed to identify the factors associated with increased suicidal risk among elderly psychiatric patients.

**Methods:**

A cross-sectional study was conducted among patients aged ≥65 years attending psychiatric consultations. Clinical, familial, and psychosocial histories were also collected. Suicidal risk was assessed using the Ducher Suicide Risk Scale, while depression, anxiety, and impulsivity were measured using standardized scales: the Hospital Anxiety and Depression Scale – Depression subscale (HADS-D), the Hospital Anxiety and Depression Scale – Anxiety subscale (HADS-A), and the Barratt Impulsiveness Scale – 11th version (BIS-11).

**Results:**

Seventy-three patients were included (male/female ratio = 1.08). According to the Ducher Suicide Risk Scale, 60% of patients showed varying degrees of suicidal ideation, from transient thoughts to active intent.

Univariate analysis revealed that higher suicidal risk was significantly associated with depressive symptoms (HADS-D, p < 0.001), anxiety (HADS-A, p = 0.003) and impulsivity (BIS-11, p < 0.001). Other significant correlates included lack of religious practice (p = 0.03), history of violence (p = 0.01) or trauma (p = 0.001), legal history (p < 0.001), family history of psychiatric disorders (p = 0.025), suicide attempts (p = 0.002), or substance use (p = 0.017). Personality disorders (p < 0.001), previous suicide attempts (p < 0.001), absence of regret following a prior attempt (p = 0.001), and current suicidal ideation (p < 0.001) were also strongly associated with elevated suicidal risk.

In multivariate analysis, lack of religious practice (p = 0.029) and depressive symptoms (HADS-D, p = 0.018) emerged as independent predictors of suicidal risk.

**Conclusions:**

Among elderly psychiatric patients, depressive symptoms and absence of religious practice are the strongest independent predictors of suicidal risk. The findings highlight the multifactorial nature of suicidality in late life, emphasizing the need for integrated screening of affective, psychosocial, and existential dimensions in geriatric psychiatric care.

**Disclosure of Interest:**

None Declared

